# Gold-iron oxide (Au/Fe_3_O_4_) magnetic nanoparticles as the nanoplatform for binding of bioactive molecules through self-assembly

**DOI:** 10.3389/fmolb.2023.1143190

**Published:** 2023-03-27

**Authors:** Elizabeth C. H. T. Lau, Michelle Åhlén, Ocean Cheung, Alexey Y. Ganin, David G. E. Smith, Humphrey H. P. Yiu

**Affiliations:** ^1^ Institute of Chemical Science, School of Engineering and Physical Sciences, Heriot-Watt University, Edinburgh, United Kingdom; ^2^ Institute of Quantitative Biology, Biochemistry and Biotechnology, School of Biological Sciences, University of Edinburgh, Edinburgh, United Kingdom; ^3^ Division of Nanotechnology and Functional Materials, Department of Material Sciences and Engineering, Uppsala University, Uppsala, Sweden; ^4^ School of Chemistry, University of Glasgow, Glasgow, United Kingdom; ^5^ Institute of Biological Chemistry, Biophysics and Bioengineering, School of Engineering and Physical Sciences, Heriot-Watt University, Edinburgh, United Kingdom

**Keywords:** iron oxide, gold, magnetite nanoparticle, insulin, cysteine, dopamine, Cys-tag, drug delivery

## Abstract

Nanomedicine plays a crucial role in the development of next-generation therapies. The use of nanoparticles as drug delivery platforms has become a major area of research in nanotechnology. To be effective, these nanoparticles must interact with desired drug molecules and release them at targeted sites. The design of these “nanoplatforms” typically includes a functional core, an organic coating with functional groups for drug binding, and the drugs or bioactive molecules themselves. However, by exploiting the coordination chemistry between organic molecules and transition metal centers, the self-assembly of drugs onto the nanoplatform surfaces can bypass the need for an organic coating, simplifying the materials synthesis process. In this perspective, we use gold-iron oxide nanoplatforms as examples and outline the prospects and challenges of using self-assembly to prepare drug-nanoparticle constructs. Through a case study on the binding of insulin on Au-dotted Fe_3_O_4_ nanoparticles, we demonstrate how a self-assembly system can be developed. This method can also be adapted to other combinations of transition metals, with the potential for scaling up. Furthermore, the self-assembly method can also be considered as a greener alternative to traditional methods, reducing the use of chemicals and solvents. In light of the current climate of environmental awareness, this shift towards sustainability in the pharmaceutical industry would be welcomed.

## 1 Introduction

Nanoparticles (10–100 nm in diameter) are suitable candidates as vehicles for the delivery of bioactive molecules (McBain et al., 2008). Their small size allows circulation inside the blood vessels while the high surface area offers a high loading capacity for bioactive cargos. Recent advancements in using nanoparticles for delivery include targeted delivery (Eskandani et al., 2022; Wu et al., 2022) and co-delivery of bioactive molecules (Hopkins et al., 2022; Li et al., 2022), aided by the development of multifunctional nanoparticles. These are particularly encouraging as they may improve both the delivery and therapeutic efficiency (Elhassan et al., 2022; Xu et al., 2022). In general, the design of a nanoparticle platform for delivery consists of a number of key features: 1) the nanoparticle core, 2) the surface functional groups, and 3) the bioactive molecules (Yiu, 2011). The core forms the main solid-state foundation for functionalities to build on. It can also possess selected key physical properties to enhance the overall properties of the nanoplatform. These properties can be magnetism (e.g., Fe_3_O_4_) (Pucci et al., 2022), fluorescence (e.g., ZnS quantum dots) (Wu and Yan, 2013), or plasmonic (Au) (Ou et al., 2021). We may also need to consider if the core needs to be (bio)degradable, depending on the desired applications (Ye et al., 2014; Qin et al., 2020). The surface functional groups, usually organic, are required to interact with the targeted cargos (e.g., drugs). It is necessary to consider what types of surface-to-cargo binding to be used. Electrostatic binding between surface functional groups and cargo is widely used because it is versatile and cargo can be released by disrupting the electrostatic bonds in presence of increased salt (or ions) content. However, more permanent binding such as covalent may also be used if the cargo needs to be retained for a longer period or going through a milieu with a high ion concentration. Most importantly, all these components must be non-toxic and safe to use *in vivo*.

There are several strategies to functionalize the nanoparticle surface with organic or inorganic methods. For inorganic nanoparticles, silanization using functional silanes such as 3-aminopropyltriethoxysilane (APTES) is commonly used to functionalize Fe_3_O_4_ and SiO_2_ nanoparticles with amine (-NH_2_) groups (Yiu et al., 2013; Kumar and Paik, 2021). Another method is to graft organic groups onto the protective coating (e.g., dextran) (Yousefvand et al., 2021; Xiong et al., 2022). However, these strategies may lead to several issues in both the synthesis and the final product characteristics/properties themselves. First of all, the added synthesis steps can only add complication to the production of the nanoparticles. These extra steps, with additional chemicals being used, may also potentially cause adverse effects in toxicity or biocompatibility, as well as further burden on sustainability in production. For example, use of solvents (e.g., toluene in a standard silanization protocol) may leave residual solvents that cause unwanted side effects on the applications. Another strategy is to use self-assembly binding between bioactive molecules and the unfunctionalized nanoparticle surface without second-step organic functionalization, circumventing the aforementioned complications.

## 2 Binding strategies *via* self-assembly

Transition metals are known to form coordination bonds with many electron donating ligands *via* their unoccupied orbitals. Indeed, this coordination chemistry has been well-developed and transition metal complexes have found wide applications in important areas such as catalysis (Crawley et al., 2022; Yadav et al., 2022) and sensing (Li et al., 2020; Ma et al., 2021). In nature, transition metal complexes are widely found in enzymes and metalloproteins (Bewley et al., 2013). For example, hemoglobin, an iron-containing metalloprotein, is probably the most well-known and abundant metalloprotein in the human body due to its vital role in the transportation of oxygen. In fact, several amino acid units, with the coordinating side chains of -NH_2_ (lysine), -COOH (glutamic acid and aspartic acid), -SH (cysteine), guanidino (arginine), and imidazole (histidine) groups, can act as ligands. Other non-amino acids bioactive molecules that can form coordination bonds with a transition metal center include dopamine and folic acid.

The formation of transition metal-ligand coordination bonds can be carried out at room temperature in an aqueous medium due to the strong affinity. This is a distinct advantage because many bioactive molecules such as proteins are solvent- and temperature-sensitive. Reactions at an elevated temperature or in an organic solvent could denature the protein molecules of interest, leading to deactivation. Regarding the transition metal center, although most transition metals can serve as the metal center for coordinating these bioactive ligands, only a few transition metal candidates can be used *in vivo* biomedical applications due to their associated toxicity. For example, it has been known for decades that Ni and Co are toxic and carcinogenic (Magaye et al., 2012). Very few safe choices of transition metals are available for scientists to develop nanoplatforms. These comprise of iron, gold, platinum, zinc, and titanium.

### 2.1 Au-thiol and Au-cysteine binding

**FIGURE 1:**
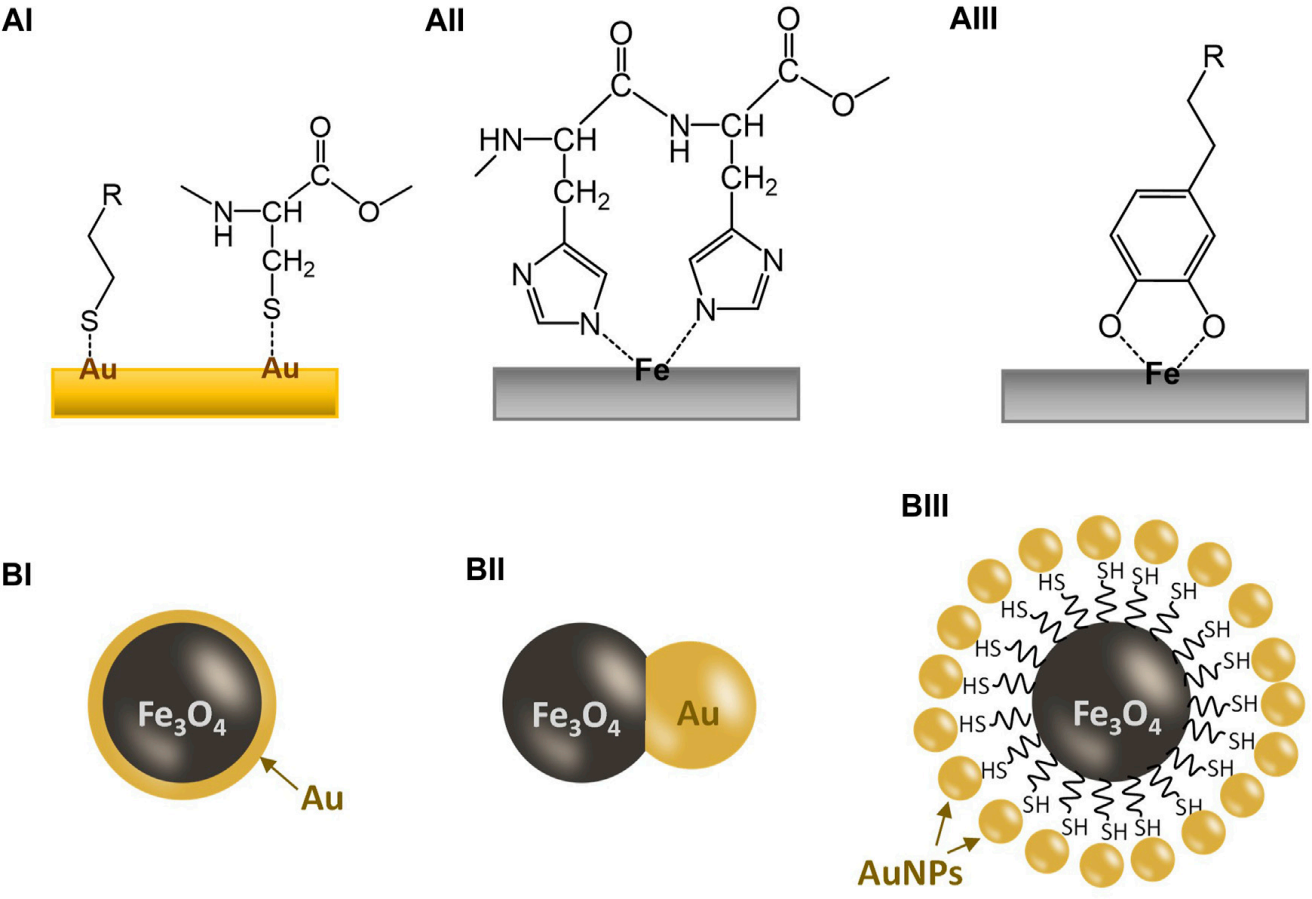
Illustration for the coordination bonding between a metal surface and a ligand and the basic designs for common Au/Fe_3_O_4_ nanoparticles. **(AI)** Interaction between a gold surface and thiol groups; **(AII)** interaction between an iron surface and a dihistidine group; **(AIII)** interaction between an iron surface and a catechol group; **(BI)** a gold-coated Fe_3_O_4_ nanoparticle; **(BII)** a Au/Fe_3_O_4_ Janus nanoparticle; **(BIII)** a gold-dotted Fe_3_O_4_ nanoparticle assembled *via* a thiol-functionalized core.

Thiolated compounds (-SH) are known to bind to noble metal surfaces (e.g., Au, Ag, Pt), due to the *π*-electron conjugation between the thiol group and the metal atoms (Figure 1AI) (Heimel et al., 2006). There is much published research in the literature studying this thiol-to-metal interaction, in particular on Au (Ulman, 2001). Au is the most popular choice of metal for this purpose because of its safety (e.g., vs. Ag), stability, and its cost (e.g., vs. Pt). There are many bioactive compounds that include thiol groups in their structure, even without modification. One important example is the cysteine unit in peptide and protein molecules, which can bind to the Au surface without modification providing that the cysteine unit(s) is accessible externally. Using protein engineering, Cys-tags can also be attached to bioactive protein molecules, enabling them for effective binding onto an Au surface (van der Meer et al., 2021). Research on using Au nanoparticles for the delivery of bioactive molecules or drugs has been widely available in the literature (Ghosh et al., 2008; Pissuwan et al., 2011). Many of these works were based on thiol- or cysteine-to-gold interactions. Recent examples include the delivery of the anticancer p53 peptides, which were bound directly to the Au nanoparticle surface *via* cysteine-to-gold interaction (Maraming and Kah, 2021).

### 2.2 Iron-dihistidine binding

Iron is another element that is widely used in biomedical research due to its safety and cost. Moreover, the magnetic properties (ferromagnetic or superparamagnetic) of some iron oxides, notably magnetite (Fe_3_O_4_) and maghemite (γ-Fe_2_O_3_), are also added advantages, including MR-contrasting (Li and Zhang, 2019), enhanced separation (Wan Ibrahim et al., 2015), targeted delivery (Palanisamy and Wang, 2019; Sharifianjazi et al., 2021), and potential for hyperthermia (Zhao et al., 2020; Pinakidou et al., 2022). Similar to many transition metals, the empty orbitals in the iron atom allow the formation of strong coordination bonds with electron-donating ligands, many of which are oxygen- or nitrogen-containing groups. One notable amino acid is histidine, which has an imidazole group on the structure, allowing coordination to transition metals (e.g., Fe, Co, Ni, Cu) (See Figure 1AII) (Kruppa and König, 2006). The histidine units in a metalloprotein can serve as a ligand to bind the metal center (Zastrow and Pecoraro, 2013; Liu et al., 2014). Indeed, the histidine-to-metal affinity has already been exploited in biotechnology, such as protein engineering, by synthetically tethering His-tags comprising six or seven consecutive histidine residues (His-6 or His-7) to maximize the binding to transition metal sites (You and Piehler, 2014). The ultimate purpose of this strategy is to facilitate the laborious protein purification process.

Despite the iron-to-histidine interaction being studied in the literature, examples of using iron oxide nanoparticles for binding His-tagged proteins are few and far between. Most scientists in this area still prefer using Ni as the transition metal for forming a chelate with His-tags due to their higher selectivity. However, as mentioned previously, nickel is considered to be toxic and unsuitable for *in vivo* biomedical applications. Currently, using unfunctionalized iron oxide nanoparticles to deliver his-tagged proteins with bioactivity is underdeveloped, but not impossible. For example, Schwaminger et al. (2019) demonstrated that unfunctionalized bare iron oxide (magnetite Fe_3_O_4_) of 12 nm can bind his-tagged GFP (green fluorescence protein) directly from the cell lysates, with 91% purity. However, in a separate study, iron (III) oxide nanoparticles were found to have the lowest affinity towards his-tagged GFP among the ferrites (Cu, Ni, Co) being tested, with CuFe_2_O_4_ showing the highest affinity (Park et al., 2018). As discussed earlier, nanoparticles with Cu, Ni and Co may not be suitable for *in vivo* applications due to their toxicity.

### 2.3 Iron-catechol binding

Iron can also form complexes with catechol ligands, notably siderophores but also including derivatives such as adrenaline and dopamine. Dopamine is an essential neuromodulator in the human body and a dysfunctional dopamine system is to be linked to the development of Parkinson’s disease (Speranza et al., 2021). Indeed, dopamine has an intrinsic affinity to iron, a relationship that is used in regulating the dopaminergic neurotransmission (Arreguin et al., 2009; Pino et al., 2017). Such affinity is so strong that dopamine can penetrate stable iron chelates for coordination to form a catechol-iron complex. Scientists have been exploiting such a naturally occurring binding phenomenon for functionalization of iron oxide nanoparticles (Xu et al., 2004), as illustrated in Figure 1AIII. Coordination of dopamine onto iron oxide nanoparticles can generate an amine surface, which is positively charged and allows further modification *via* organic reactions. However, it was also found that dopamine coordination can facilitate the degradation of iron oxide nanoparticles (Shultz et al., 2007). This degradation may also be seen as positive as a possible excretion mechanism for the spent nanoparticles.

Due to such strong bonding, catechols have also been used to stabilize iron oxide nanoparticles. For example, Amstad et al. (2009) demonstrated the binding of several catechol-modified PEG (5 kDa) onto iron oxide nanoparticles for enhancing colloidal stabilization (Zvarec et al., 2013). Similarly, a catechol-modified chitosan has also been used to stabilize iron oxide nanoparticles. However, in cases of using catechols for nanoparticle stabilization, the bioactive molecules to be delivered will need to be attached onto the stabilizing agents (e.g., PEG or chitosan) *via* further functionalization.

## 3 Gold-iron oxide (Au/Fe_3_O_4_) nanoparticles

Among the aforementioned transition metal nanoplatforms, gold and iron oxide nanoparticles are by far the most popular choices in research for *in vivo* biomedical applications. Both are considered to be of low cytotoxicity. While Au can enhance CT-imaging (Bao et al., 2022) and ultrasound imaging (Li et al., 2018), iron(II,III) oxide, or magnetite, nanoparticles had been widely used for MR contrasting (Olariu et al., 2011; Yiu et al., 2012; Yue et al., 2022) due to their superparamagnetic property. In addition, magnetic iron oxide nanoparticles (including maghemite, *γ*-Fe_2_O_3_) have also been tested for targeted drug delivery (Kim et al., 2020) and hyperthermia (Wang et al., 2022), using an external magnetic field. Therefore, nano-composite hybrids of these two components would lead to the development of “multi-functional” nanoplatforms (Leung et al., 2012).

### 3.1 Gold-coated iron oxide nanoparticles

Gold-coated iron oxide magnetic nanoparticles have been reported as early as in 2001 (Lin et al., 2001; Zhou et al., 2001). Initially, the gold coating served as a protective coating for the magnetic core, as well as an active layer for attaching bioactive molecules, as illustrated in Figure 1BI. However, even to date, not many reports on Au-coated iron oxide nanoparticles for biomedical applications have provided conclusive evidence, e.g., TEM images, that the magnetic cores were completely encapsulated in a non-porous gold coating (e.g., Hoang et al., 2022). Indeed, such a continuous but thin gold coating is difficult to achieve. This is because the deposition mechanism of gold is mostly linked to the precipitation from [AuCl_4_]^−^ salt by increasing the pH (using a base such as NH_3_ or NaOH) of the suspension of magnetic iron oxide nanoparticles. This tends to form gold clusters at a nanoscale on the iron oxide nanoparticle surface rather than a thin layer of coating. Another difficulty arises as these gold nanoclusters cannot always be easily identified using conventional analytical techniques such as XRD and TEM if the dimension of the Au coating is less than a few nanometers. Small non-crystalline clusters showed very broad peaks with low intensity in XRD while the EDX (elemental mapping) function of TEM does not have such a high resolution at a few nanometers scale. Therefore, many of these reported “gold-coated magnetic iron oxide” nanoparticles reported in the literature are essentially composite with gold deposited on an iron oxide host as nanoclusters. Non-etheless, these composites do possess the critical chemical and physical properties of both gold and iron oxide nanoparticles, allowing these nanoplatforms to perform specific tasks that they were designed for. Li et al. (2014) showed a detailed analysis of an Au-coated Fe_3_O_4_ nanoparticle sample at a high resolution and demonstrated its use in NIR-triggered drug delivery for cancer therapy. However, in order to adapt to the NIR-responsiveness, a complex coating is required for drug release.

### 3.2 Gold-iron oxide Janus nanoparticles

Janus nanoparticles are dumbbell-like particles composed of two-halves of different components, in this case, gold and iron oxide, as shown in Figure 1BII. They are usually formed by growing the second component (usually gold) on the surface of a particle of the first component (iron oxide) under a controlled manner. However, controlling such growth so that a true Janus nanoparticle is to form can be difficult. There are dozens of reports on gold-iron oxide Janus nanoparticles since the early 2010s but only a few reported particles are of verified dumbbell morphology (Song et al., 2017; Liu et al., 2019; Efremova et al., 2021).

Moreover, most of these reports on gold-iron oxide Janus nanoparticles are prepared for multimodal imaging with enhanced contrasting properties (e.g., *via* plasmonic resonance) (Chen et al., 2017; Liu et al., 2019). Applications of these Janus nanoparticles as nanoplatforms for the delivery of bioactive molecules are not widely reported. One possible reason is that the morphology of a Janus object does not give a high surface area-to-volume ratio of gold. Therefore, it does not help to improve their binding capacity. If the gold components are dispersed on the iron oxide surface as small clusters, the amount of gold on surface is maximized, leading to a higher binding capacity of the bioactive molecules.

### 3.3 Gold-dotted iron oxide nanoparticles and other examples

To maximize the gold surface for binding thiolated or cysteine species, gold-dotted iron oxide nanoparticles are developed. There are two main designs; gold nanoparticles impregnated directly onto the iron oxide surface and gold nanoparticles bound onto a thiolated surface of a thiol-functionalized iron oxide nanoparticle (as seen in Figure 1BII). The resultant materials have little difference in terms of ability to bind thiolated compounds but direct impregnation is a simpler procedure to prepare these gold-dotted iron oxide nanoparticles. Zhao et al. demonstrated the synthesis of “strawberry-like” Au-dotted magnetic nanoparticles for CT-MR dual-contrast imaging *via* a thiolated magnetic nanoparticle as the core (Zhao et al., 2015). The nanoparticles were also embedded with a fluorescence property, allowing a powerful diagnosis for liver diseases. Indeed, biomedical imaging and diagnosis using Au/Fe_3_O_4_ nanoparticles is a well-established area of research. For example, Hu et al. (2016) reported an Au/Fe_3_O_4_ “nanostar” structure for multimodal imaging while Wang et al. (2016) used a “nanocage” Au/Fe_3_O_4_ structure to perform T_1_-T_2_ dual MRI diagnosis for tumours. In all these examples, the Au/Fe_3_O_4_ nanostructures were shown to be biocompatible.

## 4 Case study

**FIGURE 2:**
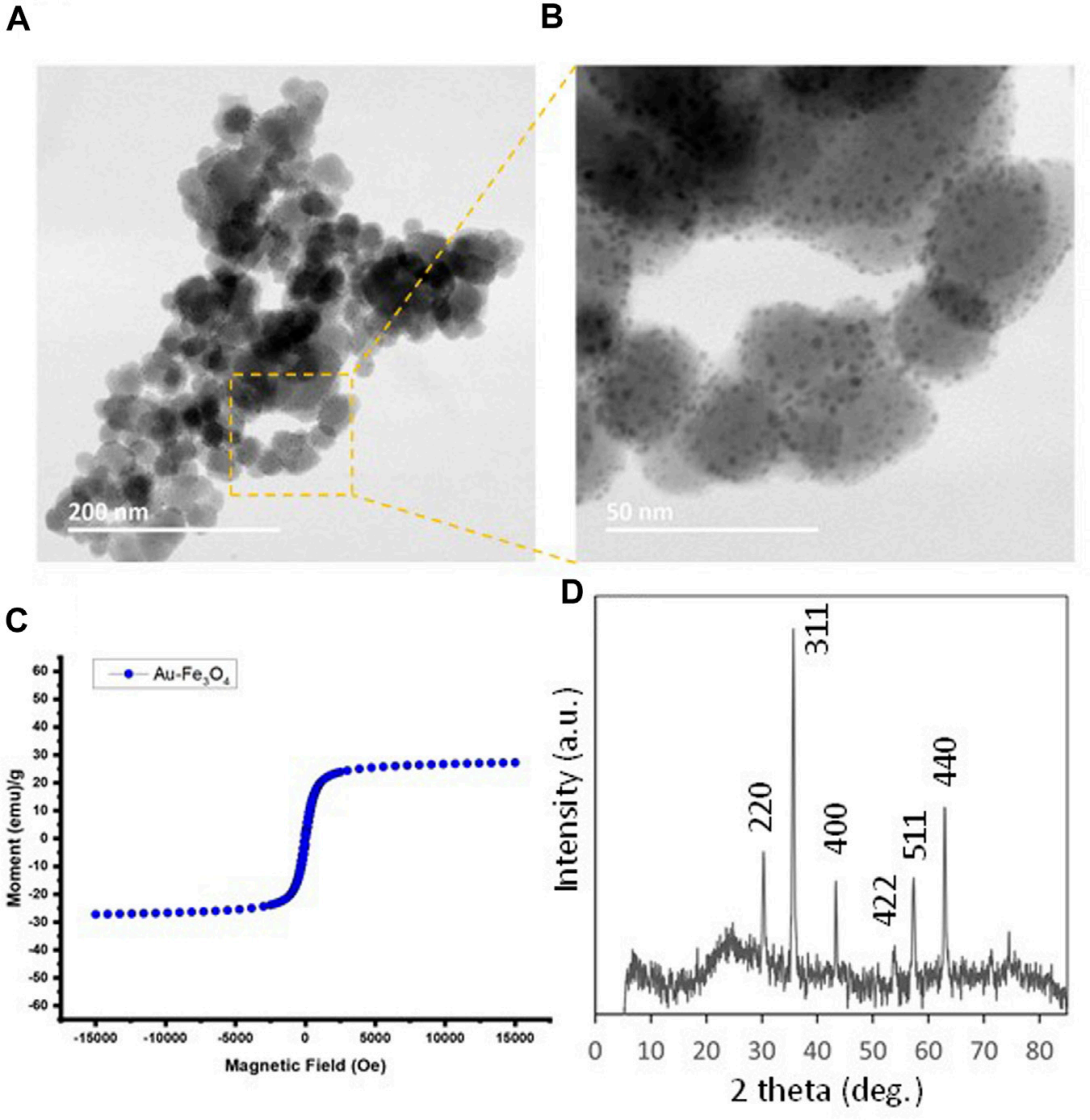
**(A, B)** TEM images for Au-dotted Fe_3_O_4_ (10%) nanoparticles prepared by impregnation of gold. **(C)** Magnetization curve (M vs. H plot) of Au-dotted Fe_3_O_4_ (10%) nanoparticles at 298K from a SQUID measurement. **(D)** XRD powder diffraction pattern for Au-dotted Fe_3_O_4_ (10%) nanoparticles, showing predominantly Fe_3_O_4_ peaks.

Previously we have reported gold-dotted iron oxide (Au/Fe_3_O_4_) nanoparticles that possessed MR-CT-ultrasound trimodal contrasting capacity (Kuhn et al., 2020). More importantly, these Au/Fe_3_O_4_ nanoparticles were found to have low toxicity *in vitro* to MCF-7 breast cancer cell lines, paving way for being developed as a delivery nanoplatform for small molecules *in vivo*. Figure 2 shows the key characterizations for an Au/Fe_3_O_4_ nanoparticle (10% w/w Au) sample. In short, the core Fe_3_O_4_ nanoparticle is around 30 nm in diameter with gold “dots” of around 2 nm dispersed on the surface (Figures 2A, B). The XRD result (Figure 2C) is consistent with a typical Fe_3_O_4_ diffraction pattern (JCPDS Card No. 019-0629). The SQUID magnetometry in Figure 2D shows a superparamagnetic character with a magnetization in saturation of 27 emu.g^−1^ at 15 kOe (See Supplementary Information for experimental procedures).

We have chosen two small peptide molecules, insulin and oxytocin, as model biomolecules, both of which are peptides having cysteine residues and, hence, suitable for direct binding to the Au component on Au/Fe_3_O_4_ surface. It was found that the binding capacities were 0.47 mg g^−1^ for insulin and 0.91 mg g^−1^ for oxytocin, which are comparable values to many nanoplatform systems in the literature.

We also carried out further investigations on the surface binding of insulin using zeta potential measurements. Au/Fe_3_O_4_ (10%) suspended in PBS showed a strong negatively charged surface (−26.7 mV) due to the surface-bound phosphate ions. When insulin was bound onto the surface, the zeta potential reduced to −12.3 mV, showing that insulin replaced the weakly bound phosphates. We also used dopamine to deactivate the Fe_3_O_4_ surface on the Au/Fe_3_O_4_ sample, reducing the zeta potential to −21.0 mV. Using this dopamine-Au/Fe_3_O_4_ sample for insulin binding, a zeta potential of −17.0 mV was obtained, suggesting that the phosphate bound on the Au surface was being replaced by insulin. This also suggested that insulin can also bind on the Fe_3_O_4_ surface, which is consistent with Schwaminger’s work (Schwaminger et al., 2015).

In order to ensure the safe use of nanoparticles and their sustainability in production, benign chemicals should be considered. For example, in this synthesis, urea was chosen as a mild base for Au deposition. This method is adapted from the preparation of metal-on-oxide supported catalysts. Therefore, scaling up is possible (up to several g per batch in laboratories), making such delivery platforms more suitable for use in the public health sector, compared with some systems reported in the literature where batch size can be limited to below 100 mg. The use of sonication allows an even dispersion of Au nanoparticles on the iron oxide surface, with a narrow particle size distribution. Also because of the nature of catalyst preparation, this method can also be adapted tois also adaptable for preparering a widehile range of metal/-support combinations, e.g., Pt/ZnO, when other properties of nanoparticles are required for specific applications.

However, there are only a small number of metal-to-organic interactions that have been exploited for self-assembly for biomedical applications. As mentioned previously, one reason is that the potential toxicity of components limits the choice of transition metals and organic groups that can be used. Modification of the small bioactive molecules should also be carefully considered as it could deactivate these molecules, causing them to lose their primary functionalities.

Compared with small bioactive peptides, binding and delivery of larger bioactive peptides may be more straightforward. First, as the peptide molecules get larger, there are a larger number of amino acid units on the surface of the peptide that may be active for binding onto the nanoparticle surface. Moreover, the orientation of these large peptides on the nanoparticle surface can also be manipulated by protein engineering, including the addition of N-terminal or C-terminal His-tags or Cys-tags, similar to the strategy that is commonly used for protein/enzyme purification. As such the exposure of active sites on the peptide can be maximized. However, adding His-tags or Cys-tags to small peptide molecules may not always be desirable as these tags may alter the fundamental characteristics significantly to a small peptide with a much shorter amino acid sequence.

## 5 Conclusion

Nanoplatforms for the delivery of bioactive molecules could be a breakthrough for nanomedicine and much advancement can be seen in the past two decades. Whilst many reports have focused on the (multi-) functionalization of the nanoparticle surface *via* organic reactions, self-assembly of bioactive molecules on purely inorganic nanoplatforms allows for simpler materials preparation, enhancing process sustainability of manufacturing, as additional organic reactions will increase the use of organic chemicals and solvents. The self-assembly method is based on the formation of coordinating bonds between the bioactive molecules and the transition metal sites on the surface of the nanoparticles. Notable candidate pairings include Au-thiol, Fe-dihistidine, and Fe-catechols. As such, an Au/Fe nanoplatform can be developed for dual delivery, if the cargos carry suitable functionalities for self-assembly, and that is where the challenges lie. Large protein molecules can carry a Cys-tag or His-tag through protein engineering, delivery of smaller peptide molecules using this method may be more restrictive. The addition of Cys-tag or His-tag could alter the primary functions of these molecules, hence, careful evaluation of peptide modifications and peptide-carrier combinations will be necessary steps in development. Using insulin and oxytocin as model molecules, we also demonstrate that Au/Fe_3_O_4_ nanoparticles can carry 0.47 and 0.91 mg g^−1^ of these bioactive peptides respectively *via* the self-assembly mechanism. In conclusion, this self-assembly route is worth exploring if the cargo molecules carry groups that can form coordination bonds with the nanoplatforms.

## Data Availability

The original contributions presented in the study are included in the article/Supplementary Material, further inquiries can be directed to the corresponding author.
