# Supplementary material for: Gold-iron oxide (Au/Fe3O4) magnetic nanoparticles as the nanoplatform for binding of bioactive molecules through self-assembly
Source: Front Mol Biosci. 2023 Mar 27;10:1143190. doi: 10.3389/fmolb.2023.1143190 (PMC10083301; doi:10.3389/fmolb.2023.1143190)
Supplement: Supplementary file 1 [file Table2.DOCX]

Supplementary Material

Gold-iron oxide (Au/Fe_3_O_4_) magnetic nanoparticles as the nanoplatform for the delivery of bioactive molecules through self-assembly

Elizabeth C. H. T. Lau, Michelle Åhlén, Ocean Cheung, Alexey Y. Ganin, David G. E. Smith, Humphrey H. P. Yiu*

*** Correspondence:** Humphrey H. P. Yiu: h.h.yiu@hw.ac.uk

# Supplementary Information on Experimental Methods

## Preparation of Au/Fe_3_O_4_ nanoparticles

A detailed procedure for preparing Au/Fe_3_O_4_ nanoparticles (Au/Fe weight ratio = 0.19) can be found in the literature (Kuhn et al., 2020). In brief, 50 mg chloroauric acid trihydrate (HAuCl_4_·3H_2_O, Alfa Aesar) were dissolved in deionised water (1 mL), and added to a suspension of Fe_3_O_4_ nanoparticles (Alfa Aesar, nanopowder, 20-30 nm) at 300 mg in 24 mL of deionised water. The reaction mixture was then homogenised in an ultrasonic bath (Fisher). Urea (Fisher) was dissolved in 5 mL of deionised water and the solution was then added drop-wisely to the Au/Fe_3_O_4_ nanoparticle suspension under sonication. The temperature of the ultrasonic bath was then increased to 80°C at a heating rate of 2°/min and this suspension was sonicated for a further 6 h at 80°C. The solid samples were then separated using a NdFeB magnet and washed using deionised water for a minimum of 10 times.

## Characterisation of Au/Fe_3_O_4_ nanoparticles

Au/Fe_3_O_4_ nanoparticles were characterised using Transmission electron microscopy (TEM), powder X-ray diffraction (PXRD), and SQUID (superconducting quantum interference device) magnetometry. In collaboration with the Kelvin Nanocharacterisation Centre at the University of Glasgow, TEM was carried out using a Tecnai microscope operated at 120 kV. For sample preparation, 20μl of a sample suspension in ethanol was drop-casted on a copper grid with carbon supporting film and then air-dried. Powder X-ray diffraction (PXRD) patterns were recorded using a Bruker D8 ADVANCE diffractometer with Cu K*α*_1_ radiation and a step size of 0.01°. The magnetic property of the sample was measured on a Quantum Design MPMS3 SQUID magnetometer equipped with a 7 T DC magnet. In a typical experiment a sample was weighed into a gel capsule. A small drop of Eicosene (Sigma-Aldrich) preheated to 60°C was added to the capsule to prevent the sample from moving within strong magnetic fields. The capsule then was weighed again to account for the mass of Eicosene.

## Binding of insulin and oxytocin onto Au/Fe_3_O_4_ nanoparticles

In a typical experiment a 2 mg/mL stock solution of Au/Fe_3_O_4_ MNPs (10 %) was sonicated and dispersed in PBS buffer alongside a 2 mg/mL solution of inuslin or oxytocin. After full dispersion 1 mL of each was added to a 2 mL Eppendorf and placed on a rotary mixer at room temperature for 2 hours. Upon completion the Au/Fe_3_O_4_ MNPs were harvested using a NdFeB magnet and the supernatant was quantified using Bradford assay to guage the binding of insulin and oxytocin.

## Bradford assay and zeta potential measurements

Bradford assay was used to quantify the amount of protein (insulin or oxytocin) bound to the surface of the nanoparticles. The assay was carried out by making BSA (bovine serum albumin) solutions of 10, 20, 30, and 40 μg/mL in PBS buffer. In a 1 mL cuvette, 20 μL of each solution was mixed with 1000 μL of Bradford reagent and incubated at 25°C for 5 minutes. The absorbance for each solution was measured in a Shumadzu UV mini 1240 spectrophotometer at 595 nm in a 1 mL cuvette (light path of 1 cm). This calibration curve was then used to determine the amount of protein bound to the sample of nanoparticles by measuring the supernatant after binding.

The electrophoretic mobility of the nanoparticles was determined with a Malvern Instruments ZetaSizer NanoZS (Malvern, UK) using a dip cell and the subsequent ζ potentials were obtained using the Smoluchowski equation. The samples were measured before and after binding with bioactive agents (insulin and dopamine).

# Supplementary Tables

**Table 1.**  Zeta potential measurements for Au/Fe_3_O_4_ with/without insulin and dopamine. All suspensions were prepared in PBS pH = 7.4

| Sample | Measurement | Zeta potential (ζ, mV) |
| --- | --- | --- |
| 10% Au/Fe_3_O_4_ | 1 | -29.1 ± 13.7 |
|  | 2 | -25.6 ± 11.4 |
|  | 3 | -25.3 ± 10.7 |
| 10% Au/Fe_3_O_4_ + Insulin | 1 | -12.2 ± 13.2 |
|  | 2 | -12.2 ± 15.5 |
|  | 3 | -12.6 ± 18.2 |
| 10% Au/Fe_3_O_4_ + Dopamine | 1 | -20.3 ± 9.5 |
|  | 2 | -20.8 ± 11.8 |
|  | 3 | -22.0 ± 9.7 |
| 10% Au/Fe_3_O_4_ + Dopamine + Insulin | 1 | -16.1 ± 9.9 |
|  | 2 | -17.0 ± 11.0 |
|  | 3 | -17.8 ± 12.5 |

# Reference

Kuhn, J., Papanastasiou, G., Tai, C. W., Moran, C. M., Jansen, M. A., Tavares, A. A., et al. (2020). Tri-modal imaging of gold-dotted magnetic nanoparticles for magnetic resonance imaging, computed tomography and intravascular ultrasound: an in vitro study. *Nanomedicine*, 15, pp. 2433–2445. doi: 10.2217/nnm-2020-0236
